# Biotechnological potential of salt tolerant and xerophilic species of *Aspergillus*

**DOI:** 10.1007/s00253-024-13338-5

**Published:** 2024-11-19

**Authors:** István Pócsi, Jan Dijksterhuis, Jos Houbraken, Ronald P. de Vries

**Affiliations:** 1 Department of Molecular Biotechnology and Microbiology, Institute of Biotechnology, Faculty of Science and Technology, University of Debrecen, Egyetem tér 1., 4032 Debrecen, Hungary; 2 HUN-REN-UD Fungal Stress Biology Research Group, Debrecen, Egyetem tér 1., 4032 Debrecen, Hungary; 3 Food and Indoor Mycology, Westerdijk Fungal Biodiversity Institute, Uppsalaan 8, 3584 CT Utrecht, The Netherlands; 4 Fungal Physiology, Westerdijk Fungal Biodiversity Institute, Uppsalaan 8, 3584 CT Utrecht, The Netherlands

**Keywords:** Aspergilli, Enzyme production, Secondary metabolites, Mycotoxins, Bioremediation, Biosolubilization, Climate change

## Abstract

**Abstract:**

Xerophilic fungi occupy versatile environments owing to their rich arsenal helping them successfully adapt to water constraints as a result of low relative humidity, high-osmolarity, and high-salinity conditions. The general term xerophilic fungi relates to organisms that tolerate and/or require reduced water activity, while halophilic and osmophilic are applied to specialized groups that require high salt concentrations or increased osmotic pressure, respectively. Species belonging to the family *Aspergillaceae*, and especially those classified in *Aspergillus* subgenus *Aspergillus* (sections *Restricti* and *Aspergillus*) and *Polypaecilum*, are particularly enriched in the group of osmophilic and salt-tolerant filamentous fungi. They produce an unprecedently wide spectrum of salt tolerant enzymes including proteases, peptidases, glutaminases, γ-glutamyl transpeptidases, various glycosidases such as cellulose-decomposing and starch-degrading hydrolases, lipases, tannases, and oxidareductases. These extremophilic fungi also represent a huge untapped treasure chest of yet-to-be-discovered, highly valuable, biologically active secondary metabolites. Furthermore, these organisms are indispensable agents in decolorizing textile dyes, degrading xenobiotics and removing excess ions in high-salt environments. They could also play a role in fermentation processes at low water activity leading to the preparation of daqu, meju, and tea. Considering current and future agricultural applications, salt-tolerant and osmophilic Aspergilli may contribute to the biosolubilization of phosphate in soil and the amelioration salt stress in crops. Transgenes from halophile Aspergilli may find promising applications in the engineering of salt stress and drought-tolerant agricultural crops. Aspergilli may also spoil feed and food and raise mycotoxin concentrations above the permissible doses and, therefore, the development of novel feed and food preservation technologies against these *Aspergillus* spp. is also urgently needed. On the other hand, some xerophilic Aspergilli have been shown to be promising biological control agents against mites.

**Key points:**

• *Salt tolerant and osmophilic Aspergilli can be found in versatile environments*

• *These fungi are rich resources of valuable enzymes and secondary metabolites*

• *Biotechnological and agricultural applications of these fungi are expanding*

## Introduction

Cellular properties like xerophily, osmophily, and halophily share a common characteristic namely low water availability/activity (Grant 2004; Kim et al. 2014; Stevenson et al. 2015). By definition, xerophilic and xerotolerant species can tolerate or reproduce under low water activity values (*a*_w_ < 0.85, according to the definition of Pitt and Hocking 2022), osmophilic and osmotolerant species can tolerate water constraints when growth media contains high amounts of solutes, and halophilic and halotolerant species can grow when a high salt, most often NaCl, concentration is present (Coleine et al. 2022). These terms seem to be flexible and often overlapping; e.g., xerophilic fungi are sometimes also called osmophilic (Grant 2004) and halophilic species may also be xerophilic (Kanekar and Kanekar 2022). Further proposals for actualization and fine-tuning of these classification terms for yeasts (Dakal et al 2014) and filamentous fungi including *Aspergillus* and *Penicillium* spp. (Kujović et al. 2024) can be found in the literature. In this review, we use the terminology found in the cited original publications to describe the cellular properties (xerophilic, osmophilic, and halophilic) of *Aspergillus* strains to avoid controversy with previous literature.

Fungi thriving well in the presence of high concentrations of chaotropic salts such as MgCl_2_, CaCl_2_, and NaBr (destabilizing) or kosmotropic salts like NaCl, KCl, and MgSO_4_ (stabilizing) can be regarded as chaophilic and chaotolerant or kosmophilic and kosmotolerant species, respectively (Zajc et al 2014; Moreno-Perlin et al. 2023). In addition, alcohol is chaotropic and the use of chaotropic *S. cerevisiae* strains is discussed in the light of biofuel production (Cray et al. 2015).

**Fig. 1:**
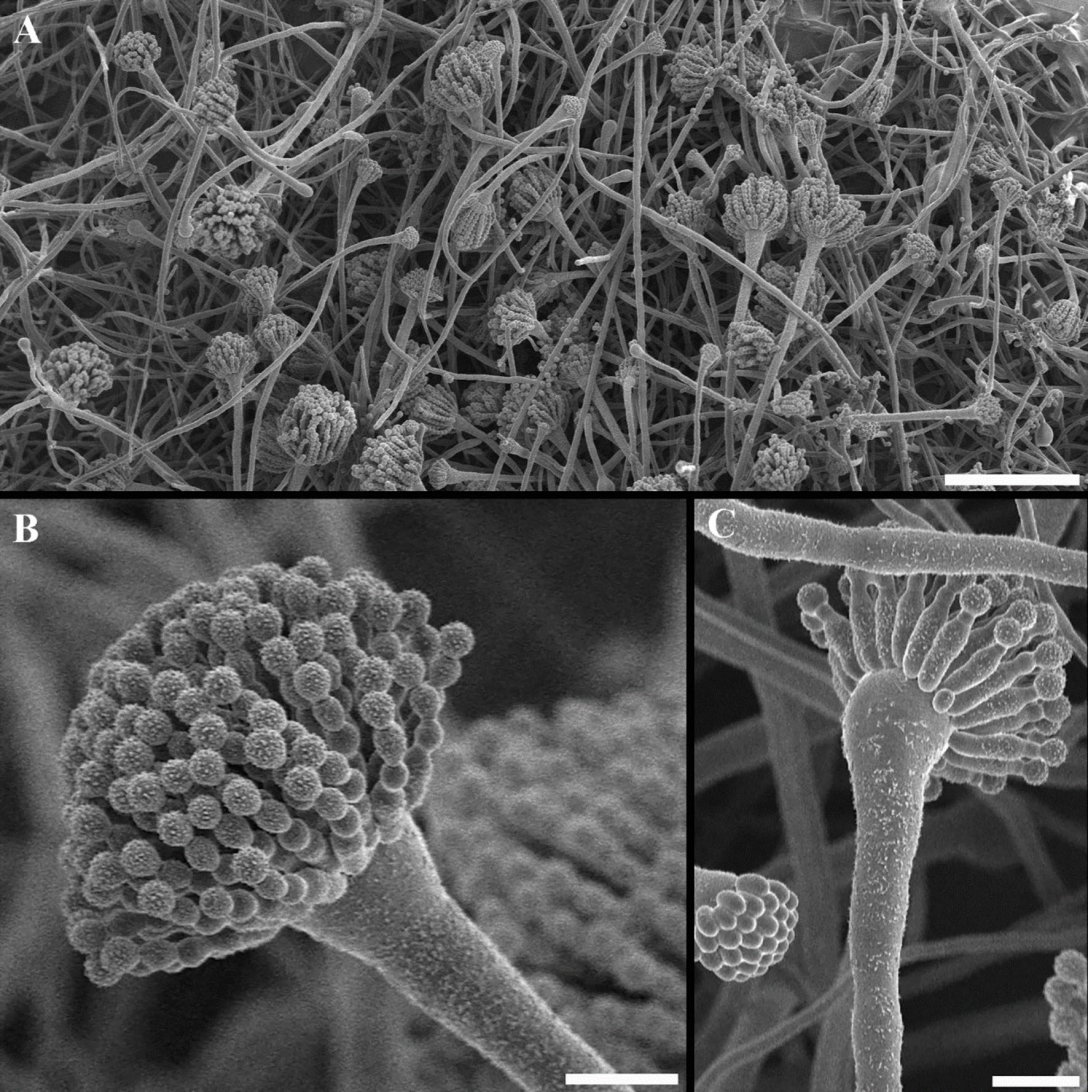
Cryo-electron scanning electron microscopy micrographs of *Aspergillus penicillioides*, one of the most xerophilic fungi known. **A** A survey of the rim of a colony, showing many conidiophores in different stages of development. **B** Detail of a conidiophore showing the formation of short rows of ornamented conidia on phialidic cells. The first conidium is formed at the base of the row. No ornamentation has developed on the cell wall during these initial stages. **C** The phialidic cells from a different angle with one or two conidia formed. There is some material present on the stipes. To the left a young conidiophore is visible on with early phialides forming. Bars are 100 µm (**A**) and 10 µm (**B**, **C**)

Not surprisingly, fungi are prominent members of many extreme ecosystems (Coleine et al. 2022), and the theoretical limits for the filamentous growth of the extreme xerophilic *Aspergillus penicillioides* (see Fig. 1) and *Xeromyces bisporus* were set to 0.632 and 0.636 *a*_w_, respectively (Stevenson et al. 2015). Furthermore, *A. penicillioides* cell division (formation of septate germlings) was detected at *a*_w_ = 0.585 with an *a*_w_ = 0.565 theoretical water-activity minimum for germination (Stevenson et al. 2017a). Xerophilic *Aspergillus* and *Xeromyces* strains can even tolerate well the high chaotropicity of glycerol-only media with concentrations in the range of 7.65 M (*a*_w_ = 0.644; chaotropic activity 20.88 kJ kg^−1^), which prevented the mycelial growth of the tested xerophilic species (Williams and Hallsworth 2009; Stevenson et al. 2017b). In accordance with these findings, *Aspergillus wentii* grew at glycerol concentrations ≤ 6.3 M (*a*_w_ = 0.708) (de Lima Alves et al. 2015). The fungus* Aspergillus halophilicus* is described to grow down to 0.654 and related to growth in libraries (Pole et al. 2017; Micheluz et al. 2022).

Importantly, the ecological significance of xerophilic species is expected to increase with progressing climate change due to more frequent incidences of drought (Coleine et al. 2022), and the biotechnological application of these species as well as their enzyme and secondary metabolite products is flourishing (Musa et al. 2018; Ibrar et al. 2020; Śliżewska et al. 2022; Hmad and Gargouri 2024).

*Aspergillus*, and especially its subgenus *Aspergillus* comprising the sections *Aspergillus* and *Restricti*, accommodates many ecologically and industrially important xerophilic and halophilic species (Chen et al. 2017; Sklenář et al. 2017; Houbraken et al. 2020). This review therefore focuses on these extremophilic Aspergilli and their biotechnological exploitability.

## Taxonomy and ecology of xerophilic fungi

### Which Aspergilli are xerophilic?

**Fig. 2:**
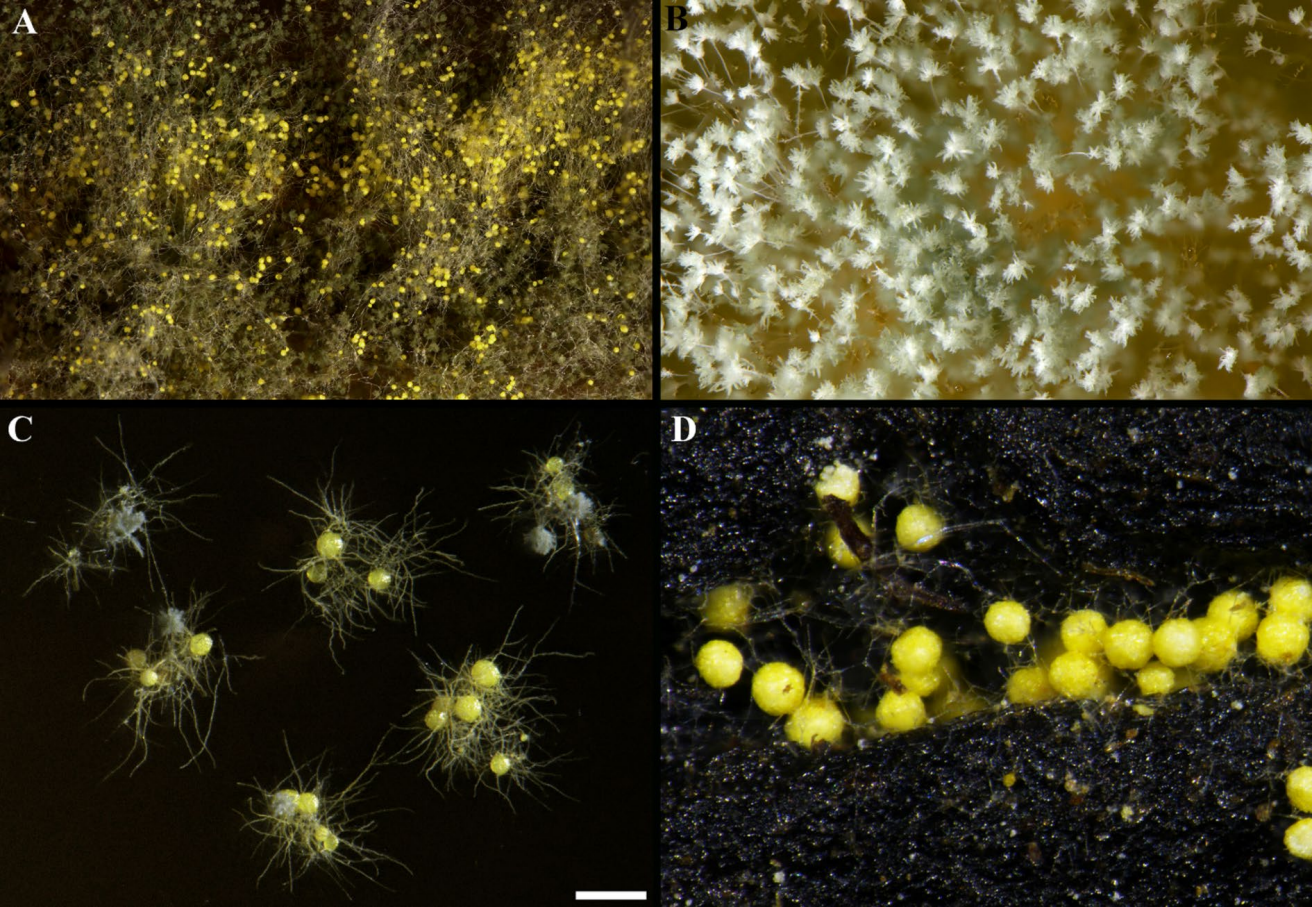
Xerophilic Aspergilli (eurotium morph). **A** *Aspergillus* sp. isolated from cured ham, and showing the characteristic cord-like growth also observed on the surface of ham. **B** The young *Aspergillus* heads that form conidia from the strain depicted in **A**, the individual rows of conidia are visible. **C** *A. chevalieri* isolated from poultry feed showing small colonies with some conidiophores, a number of ascomata containing ascospores, and a small mycelium. **D** This *A. cristatus* is growing on fermented tea leaves and some mycelium and only ascomata are visible in this condition. Bar = 500 µm

According to Geiser et al. (2006), the subclass *Eurotiomycetidae* (phylum *Ascomycota*, subclass *Pezizomycotina*, class *Eurotiomycetes*) includes many xerophiles. With the introduction of a single name nomenclature, the genus *Eurotium*, which contained many important xerophilic species, was synonymized with *Aspergillus* (Samson et al. 2014). Many *Eurotium* species are currently known under their *Aspergillus* name (Hubka et al. 2013; Samson et al. 2014; Chen et al. 2017). The genus *Aspergillus* within the order *Eurotiales*, family *Aspergillaceae*, is subdivided in subgenera and sections, and most xerophilic species are classified in subgenus *Aspergillus* and *Polypaecilum* (Fig. 2). The subgenus *Aspergillus* is further divided in two sections, *Aspergillus* and *Restricti* (Houbraken et al. 2020), and these contain the majority of xerophilic Aspergilli (Chen et al. 2017; Sklenář et al. 2017). All 32 accepted species of section *Aspergillus*, and all 22 species of section *Restricti* grow well on malt extract agar supplemented with 60% sucrose (1.75 M). These data show that the ability to grow at lowered water activity is a shared character within taxa of subgenus *Aspergillus*, and remained present during evolution.

*Aspergillus* subgenus *Polypaecilum* is sister to all other *Aspergillus* subgenera (Fig. 2). Currently, 24 species are accepted in subgenus *Polypaecilum*. The species in the subgenus differ in their ability to grow in the presence of glucose or NaCl, and contains specific halophilic and sugar-tolerant fungi. The majority of subgenus *Polypaecilum* species are able to grow on MY10-12, a medium with 10% NaCl (1.7 M) and 12% glucose. In contract, the subgenus *Polypaecilum* members *A. caninus* and *A. chlamydosporus* are unable to grow at 10% NaCl, but do grow in the presence of 40% glucose (2.2 M), while the halophile *A. salinarus* does not grow without < 5% NaCl added to the medium presence (Tanney et al. 2017; Houbraken et al. 2020). As these species are relatively close, but more distant from the other xerophilic Aspergilli, they could have their own repertoire of enzymes or other properties that could make them amenable for biotechnological use.

Outside subgenus *Aspergillus* and *Polypaecilum*, *A. candidus* (sect. *Candidi*), *A. ochraceus* (sect. *Circumdati*), and *A. wentii* (sect. *Cremei*) are reported to grow at or below 0.75 *a*_w_. *A. wentii* belongs to subgenus *Cremei* section *Cremei*; this section is a sister of sections *Restricti* and *Aspergillus* (Fig. 2). Section *Candidi* includes nine accepted species (Glassnerova et al. 2022), and details on their minimal water activity for growth are lacking. However, these species are reported to produce interesting enzymes. There is a limited number of Aspergilli reported that grow at water activities between 0.75 and 0.8. In the original definition of xerophilic fungi by Pitt (1975), growth below 0.85 *a*_w_ was used as the threshold. Here, we use 0.80 *a*_w_ as threshold, because otherwise a large number of Aspergilli would be included, as many can grow up to 0.85 *a*_w_. *Aspergillus* species that have their minimum water activity between 0.75 and 0.80 *a*_w_ include *A. terreus* (sect. *Terrei*), *A. flavus*, *A. tamarii* (sect. *Flavi*), *A. sydowii*, *A. versicolor* (sect. *Nidulantes*), and *A. niger* (sect. *Nigri*) (Segers et al. 2015; Pitt and Hocking 2022). Phenotypically similar species are classified within each section mentioned above (e.g., *Candidi*, *Circumdati*, *Flavi*, *Nigri*, *Terrei*). The minimal water activity of those phenotypically similar species is not studied, but it is likely that some of them will have similar abilities to grow below 0.80 *a*_w_.

### Natural appearance of xerophilic Aspergilli

Xerophilic *Aspergillus* spp. have also been collected in deserts (Veana et al. 2014a, b; Moreno-Perlin et al. 2023), polar deserts (de Menezes et al. 2021), saline soils (Radwan et al. 1984), and high-altitude soils (Petrovic et al. 2000). Hypersaline water environments like salterns, salt lakes, and hypersaline stream water are in general rich resources of xerophilic/halophilic fungi including *Aspergillus* spp. (some of them are formerly named *Eurotium*) (Butinar et al. 2005, 2011; Zajc et al. 2012; Jaouani et a., 2014; Chamekh et al. 2019; Chung et al. 2019; Diguță et al. 2019). Halophile Aspergilli are isolated from the Dead See (Nazareth et al. 2012) and marine sediments (González‑Martínez et al. 2019). Halophilic Aspergilli may be present in diverse ecological niches like *A. penicillioides* found in various athalassohaline (Dead See), thalassohaline (solar salterns), and polyhaline (estuary and mangroves) environments (Nazareth and Gonsalves 2014). Importantly, halophilic Aspergilli like *A. sydowii* EXF-12860 can survive or even slightly grow at saturated, 5.13 M NaCl (*a*_w_ = 0.75) concentration (Jiménez-Gómez et al. 2020). Moreover, xerophilic fungi including mycotoxigenic *Aspergillus* spp. are detectable on pre-harvest maize under low rainfall with dry spell weather conditions (Katati et al. 2023). As soil is a repository of an immense richness of fungal species, the dried soil must be an untapped resource of xerophilic Aspergilli. Interestingly, *Aspergillus* subgenus *Polypaecilum* species are isolated from cave walls and even a saltmine (Tanney et al. 2017; Houbraken et al. 2020).

### Xerophilic Aspergilli in the human environment

Harvested, stored, and dried crops (e.g., in silo or other storage environments) reflect an important natural environment for xerophilic fungi, among then the Aspergilli, as low-water activity plant material is ubiquitous worldwide. For example,* A. candidus* is clearly xerophilic and isolated from stored foods (e.g., corn kernels, rice and often isolated from nuts, processed meats), and improperly stored commodities and ingredients (Houbraken et al. 2020), and is reported to grow to 0.75 water activity (Pitt and Hocking 2022). Osmophilic fungi like *A. glaucus* are isolated from stored foodgrain (Rao and Kalyanasundaram 1983) and xerophilic Aspergilli also appear in feeds and feedstuffs (Greco et al. 2015; Dijksterhuis et al. 2019).

The xerophilic Aspergilli colonize typical human-related environments and processed foods. It is well-documented that xerophilic/halophilic fungi, including xerophilic Aspergilli, thrive in low water activity foods and (improperly stored) ingredients, leading to spoilage (Wheeler and Hocking 1988; Roessler and Ballenger 1996; Hasan 1998; Vytrasová et al. 2002; Xu et al. 2011; Mohamed et al. 2012; De Clercq et al. 2015; Xing et al. 2016; Chen et al. 2017; Buerman et al. 2019; Rodríguez-Andrade et al. 2019; Hagiuda et al. 2023; Santos de Almaide et al. 2023). However, also non-food-related human environments are of interest as xerophilic fungi can be easily collected from the dust in houses, museums, storage rooms, etc. (Samson and van der Lustgraaf 1978; Rijckaert et al. 1981; Abdel-Hafez et al. 1990; Takahashi 1997; Chen et al. 2017; Tanney et al. 2017; Hagiuda et al. 2022, 2023), bedding dust (Hashimoto et al. 2020), and dust at various work places (Gutarowska et al. 2018). In addition, these fungi contaminate paintings, deteriorate canvases and painting compounds, and disfigure books and museum objects (Polo et al. 2017; Zalar et al. 2023; Kujović et al. 2024; Bastholm et al. 2024).

Xerophilic species of section *Restricti* (e.g., *A. penicillioides*, *A. halophilicus*, *A. restrictus*) are typically isolated from house dust, seeds, sweet food products (cakes etc.), surfaces (as leather, books, textile, wood), and dried products as cereals, feed (stored in silo), or tea (Sklenář et al. 2017). The section *Aspergillus* species (e.g. *A. glaucus*, *A. proliferans*, *A. ruber*, *A. montevidensis*), formerly mostly classified in *Eurotium*, are typically isolated from air, indoor surfaces and artifacts, fermented tea and vanilla, fermented soya beans (meju), fruits and low water activity preparations, smoked ham and sausages, soil and dung, cellophane, butter, chocolate, caves, insects, kernels, cereals in silo, and some medical samples (Chen et al. 2017). *Aspergillus* subgenus *Polypaecilum* are isolated from cave walls, house dust, human ear and keratitis, a saltmine, yeast extract, fodder, and soya beans.

### Other aspects of xerophilic fungi

**Fig. 3:**
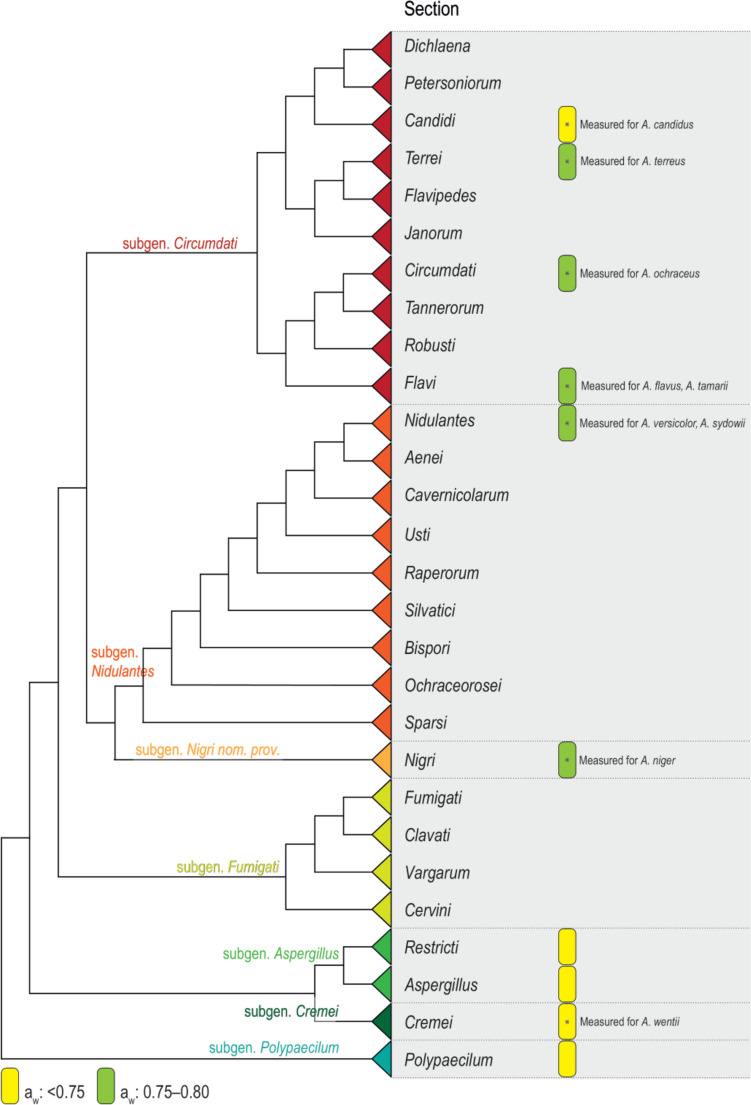
Dendrogram based on the phylograms published in Houbraken et al. (2020), Steenwyk et al. (2024), and Visagie et al. (2024) showing the phylogenetic relationship between the subgenera and sections in the genus *Aspergillus*. The occurrences of xerophilic species with tolerated water activity ranges (yellow and green rectangles) are indicated in the figure

Continuous monitoring of these molds and appropriate countermeasures to control them are needed in bakeries (Vytrasová et al. 2002), chocolate confectionery factories (De Clercq et al. 2015), dried and fermented meat and fish manufacturing plants (Scaramuzza et al. 2015; Rico-Munoz et al. 2019; Zadravec et al. 2019), and production sites of high sugar products such as confectionery, dried fruit, jams, and conserves (Hocking 2001). Xerophilic Aspergilli are also promising candidates in fermentation drinks like tea (e.g., Chinese dark “golden flower” tea produced by *A. cristatus*, Fig. 3) (Mao et al. 2017; Lu et al. 2022; Cheng et al. 2024) and other procedures that use fermentation of dried or drying material leading to the production of spirits as daqu (Hou et al. 2024) or soya-related products as meju (Yun et al. 2009). Other examples include xerophilic Aspergilli naturally growing in some food technological processes like ham and other dry cured meat production, where species such as *A. chevalieri* and *A. proliferans* can predominate (Fig. 3; de Almeida et al. 2023) and dried salted fish (Wheeler 1988). Halotolerant *Aspergillus* spp. may also appear in food processing waste like *A. olivimuriae* in olive brine (Crognale et al. 2019). Importantly, xerophilic *Aspergillus* spp. may be detected in various clinical samples (Siqueira et al. 2018), and the soil-inhabiting *Eurotium repens* (= *A. pseudoglaucus*) may also colonize the surfaces of the skin and bones of human cadavers (Ishii et al. 2006). Xerophilic fungi, due to their stress resistance in relation to water availability, may be capable to grow in low-temperature environments compared to many other *Aspergillus* species (e.g. *A. halophilicus* is reported to grow at low temperatures) and they also probably survive periods of too much drought by means of stress-resistant ascospores (Wyatt et al. 2015a, b).

## Mechanism of xerophily and salt-adaptation

There are a number of mechanisms which help xerophilic and halophilic fungi to survive under high-osmolarity and high-salinity conditions (Gunde-Cimermann et al. 2018; Coleine et al. 2022; Hmad and Gargouri 2024). These adaptation mechanisms are species-dependent and may include extracellular polysaccharide substance production, modulation of cell wall structure, composition and pigmentation, altering membrane composition and fluidity, maintaining intracellular ion and compatible solute concentrations, etc. (Gunde-Cimermann et al. 2018; Coleine et al. 2022; Gostinčar et al. 2022; Micheluz et al. 2022; Hmad and Gargouri 2024).

As far as the Aspergilli are concerned, genomic adaptation of the halophilic Dead See fungus *A. ruber* (= *E. rubrum*) to its hypersaline environment include (i) large excess of acidic amino acid residues; (ii) higher gene counts for A-/B-barrel proteins and catalases; (iii) upregulation of glycerol-3-phosphate dehydrogenase, a key enzyme in glycerol production, under high-salt stress; (iv) higher expression of genes related to ion and metabolite transport at high salinity; and (v) adjusting of cell wall components and structure (β-glucans, chitin binding) to high NaCl concentrations (Kis-Papo et al. 2014).

In the case of the extreme xerophilic fungus *Xeromyces bisporus* (family *Aspergillaceae*), low water activity (*a*_w_ 0.68) upregulated some steps of glycerol biosynthesis and genes modulating sterols, phospholipids, sphingoglycolipids, and cell wall structure were also differentially expressed (Leong et al. 2014). Furthermore, the halophilic fungus *A. sydowii* restructures and strengthens its cell wall with enhanced chitin biosynthesis and incorporation of α-glucan into the surface layer of cell wall when exposed to hypersaline conditions (Fernando et al. 2023).

The filamentous fungus model organism *A. nidulans* is not osmophilic and has been characterized with medium NaCl and relatively low-medium sorbitol tolerances among the Aspergilli depending on the strains as well as on sporulation and culture (surface agar, liquid submerged) conditions (de Vries et al. 2017; Emri et al. 2018; Orosz et al. 2018; Király et al. 2020a, b; Bodnár et al. 2023). A previous study by Beever and Laracy (1986) showed that *A. nidulans* biA1 was xerotolerant owing to its compatible solute production. In submerged liquid cultures, *A. nidulans* THS30 responded to increased osmolarity by bulk upregulation of HogA signaling as well as glycerol and trehalose metabolism genes (Bodnár et al. 2023). Importantly, similar size HogA-dependent stress response was not observable in the case of the xerophilic *A. ruber* (= *E. rubrum*; Kis-Papo et al. 2014), the halophilic *X. bisporus* (Leong et al. 2014), or the osmophilic *A. wentii* (Bodnár et al. 2023). This clearly indicates major differences in high-salt adaptations of *Aspergillus* spp. depending on the niches they occupy and also on short-term and long-term hypo- and hypersaline exposures (Kis-Papo et al. 2014).

It is tantalizing to realize that these fungi have so many cellular adaptations in place as, e.g., compatible solute accumulation and membrane adjustments, that these cells do not “feel” stress and that stress pathways are not used under conditions that other fungi would apply them. Alternatively, they could use the pathway to provide this level as it is rewired to provide protection for instance in being more constitutively expressed.

Nevertheless, functional characterization of elements and regulation of the high-osmolarity adaptation systems of osmophilic/xerophilic fungi are likely to lead to a deeper understanding of the molecular background of osmophily/xerophily in the future but our knowledge in this important field is still limited to some genes of *A. christatus*, e.g., *Achog1* (Shao et al. 2022; Liu et al. 2024), *Acpmk1* (Liu et al. 2024), and *Acmpk1* (Liu et al. 2024), putatively encoding mitogen-activated protein kinases, whose orthologs orchestrate various cellular processes including environmental stress responses in fungi (Fuchs and Mylonakis 2009; Hagiwara et al. 2016; Van Drogen et al. 2020; Liu et al. 2024). On the other hand, overexpression of *AcndtA* putatively coding for an NDT80 DNA-binding protein, whose ortholog is involved in the sexual reproduction of *A. nidulans* (Katz and Cooper 2015), adversely affected the growth of *A. cristatus* in the presence of osmolytes in comparison to the wild-type control strain (Wang et al. 2021). Therefore, further studies are urgently needed to plot the regulatory networks supporting osmophilic/xerophilic growth in these fungi especially concerning general as well as species-specific and niche-specific elements and features.

## Industry-related applications

Within the frame of this review, the following important industry-linked areas will be covered in xerophilic/halophilic *Aspergillus* research: (i) production of salt tolerant enzymes, (ii) secondary metabolite production, and (iii) bioremediation in high-salt environments by xerotolerant/xerophilic fungi, and (iv) heterologous expression of stress tolerance genes in industrial yeast and *Aspergillus* strains.

### Production of salt-tolerant enzymes

Halophilic filamentous fungi including *Aspergillus* spp. are excellent resources of various enzymes of industrial relevance such as proteases, glycosidases, and oxidoreductases (Ali et al. 2014a, 2019; Glässnerová et al. 2022; Śliżewska et al. 2022; Ibrahim et al. 2023; Ben Hmad and Gargouri 2024). NaCl is a kosmotropic salt and, therefore, it stabilizes water-biomolecule interactions, but it may also decrease catalytic activity via influencing Coulombian interactions during substrate binding and catalysis (Park and Raines 2001).

#### Proteases, peptidases, glutaminases, γ-glutamyl transpeptidases

Fungal proteases/peptidases (EC 3.4) are produced by a number of filamentous fungi including *Aspergillus* spp. and have an enormous biotechnological potential (de Souza et al. 2015). Because industrial conditions are often harsh, enzymes produced by extremophilic fungi including halophiles are considered to improve the performance of these industrial processes (Salwan and Sharma 2019). Kōji molds (Ito and Matsuyama 2021) belonging to the genus *Aspergillus* are widely used in high-salt fermentations of soybean pastes, curds, and soy sauces, and their proteolytic enzymes releasing peptides and amino acids (flavor enhancing molecules) should also tolerate NaCl at high concentrations and should have a relatively high catalytic activity (Devanthi and Gkatzionis 2019; Ito and Matsuyama 2021; Qiao et al. 2022; Gao et al. 2023).

The kōji molds *A. oryzae*, which was described as osmophilic when cultured in the presence of 2.0 M sorbitol at 37°C (de Vries et al 2017) and *A. sojae*, are excellent resources of salt-tolerant proteases, peptidases, and glutaminases (EC 3.5.1.2; key hydrolytic enzymes for flavor enhancement) (Yuzuki et al. 2015). *A. oryzae* salin-tolerant proteases can withstand NaCl concentrations up to 18% (≈ 3 M; Su and Lee 2001a; Gao et al. 2019), and have been purified and are well-characterized (Su and Lee 2001b; Gao et al. 2019).

Further research is in progress to develop new *A. oryzae* strains for soy sauce fermentation with elevated production of proteases with high salt (> 18%) and acid tolerances (Gao et al. 2023). Options to further increase production of salt-tolerant proteolytic enzyme activities in soy sauce fermentations are either the overexpression of selected genes in *A. oryzae* (e.g., salt tolerant prolyl aminopeptidase; Matsushita-Morita et al. 2010) or the heterologous expression of these genes in the salt and sugar-tolerant yeast *Zygosaccharomyces rouxii* (Yuzuki et al. 2015). The application of fungal proteases is spreading wide in various areas including food processing, detergents, textile industry, and waste treatments, which intensifies screening for and the characterization of proteases of halophilic Aspergilli, including *A. reticulatus* (Chung et al. 2022).

Among the osmophilic/xerophilic Aspergilli (de Vries et al. 2017; Takenaka et al. 2019; Király et al. 2020a), *A. glaucus* and *A. pseudoglaucus* (= *E. repens*), which are used to ferment and ripen dried tuna bonito (Katsuobushi), are rich resources of a broad spectrum of hydrolytic enzymes, among which aspartic proteases may find their industrial applications in bleaching red-pigmented heme proteins like myoglobin and hemoglobin (Aoki et al. 2013; Takenaka et al. 2017, 2019).

γ-Glutamyl transpeptidases (EC 2.3.2.2; GGTs) produced by kōji molds (*A. oryzae*, *A. sojae*) release free l-glutamic acid as an important flavor (“umami” flavor) enhancer but cannot tolerate high (≈17–18%) NaCl concentrations. Nevertheless, the xerophilic *A. sydowii* produces salt-adaptive GGT in excellent yields in solid-state fermentations with initial water activity of *a*_w_ = 0.85 (Nishikawa et al. 2022). In a most recent study (Senba et al. 2023a), a chimeric *A. oryzae*-*A. sydowii* (N-terminal region) GGT was engineered and expressed in *A. oryzae*. The chimeric enzyme showed excellent salt, acidic pH, and heat tolerance when compared to the parental enzymes and, therefore, its potential application can be considered, e.g., in brine fermentations (the second stage of soy sauce fermentation) (Senba et al. 2023a).

#### Glycosidases

Biomass-decomposing filamentous fungi are an exceptionally wealthy resource of hydrolytic enzymes, including a wide array of carbohydrate-active glycosidases (Benini 2020, Nath and Kango 2021; Chityala et al. 2023), which have been used in many biotechnological processes aimed at valorization of agro-industrial by-products (Barcelos et al. 2020). Enzymes of extremophilic fungi including halophiles are gaining ground in bio-based economy incorporating biorefineries producing, e.g., biofuels (Guerrieroa et al. 2015; Choudhary et al. 2022; Mohsin and Papageorgiou 2022). It is noteworthy that non-methylotrophic (e.g., *Saccharomyces cerevisiae*) and methylotrophic (e.g., *Pichia pastoris*) yeast-based heterologous expression systems are developing fast for the production of industrially relevant extremophile enzymes (Chityala et al. 2023). However, these systems are inferior in enzyme production levels, when compared with filamentous species, such as from *Aspergillus* and *Trichoderma* (Su et al. 2012).

Industrial production of halophilic enzymes, especially heterologous production, would preferably use already-established production hosts (e.g., *A. niger*, *A. oryzae*), but these are not species known for their high salt tolerance (see above). It is therefore crucial to determine whether halotolerant enzymes can be functionally produced in low-salt conditions, as this would be highly preferred in industrial settings. If this is the case, then the production of these enzymes is no different from production of other fungal enzymes and can use established strains, protocols, and fermentation conditions. However, should some of the halophilic enzymes only be stable when produced under high-salt conditions, a salt-tolerant host needs to be used. While this review demonstrated the presence of many salt-tolerant *Aspergillus* species, most of these have not been developed as an industrial production host. An exception to this is the production of xylanases in *A. sydowii* (Brandt et al. 2020), but it was not studied whether these enzymes were halophilic. A recent review provided an overview of fungal halophilic enzymes (Hmad and Gargouri 2024) and their industrial applications. Many of the enzymes discussed in that review prefer high-salt media for their production, as they are more soluble in the presence of salts and have a smaller number of hydrophobic residues, an acidic iso-electric point, and an abundance of negative charge on their surface (Hmad and Gargouri 2024).

For example, the unique, thermostable, pH stable, and salt-tolerant (up to 4.0 M NaCl) GH5 cellulase (“AgCMCase”) produced by *A. glaucus* CCHA was purified, characterized, and expressed in *P. pastoris* (Li et al. 2018). This cellulase is effective in releasing reducing sugars such as glucose and cellobiose from corn and rice straw, and hydrolysis parameters for GH5 cellulase were optimized by response surface methodology (Chen et al. 2020). Furthermore, acidophilic and halophilic β-glucosidases homologously expressed in marine *A. niger* using the *A. nidulans* glyceraldehyde 3-phosphate dehydrogenase (*gpdA*) constitutive promoter may find their future applications in the degradation of lignocellulosic biomass (Cai et al. 2019). These β-glucosidases hydrolyze cellobiose effectively and, hence, alleviate the inhibition of cellulose-degrading endoglucanases by this disaccharide (Cai et al. 2019).

The moderate halophilic *A. caesiellus* H1 strain isolated from a sugarcane bagasse fermentation produced thermostable and halostable cellulase activity together with xylanases, manganese peroxidase, and esterases, underlining the potential of this *Aspergillus* sp. in lignocellulose-based biotechnological processes (Batista-García et al. 2014). Hopefully, a highly salt-tolerant cellulase produced by the halophilic *A. terreus* UniMAP AA-6 strain can be used in in situ saccharification of ionic liquid pretreated lignocellulose biomass (Gunny et al. 2014). Furthermore, a cellulase from the halophilic *A. flavus* TISTR 3637 may be applicable in the conversion of alkaline-pretreated biomass into glucose in bioethanol production (Bano et al. 2019). It is noteworthy that the activity of the salt-tolerant cellulase cocktail produced by a marine *A. niger* strain was improved via overexpression of selected cellulase genes and mixed recombinant strain fermentations (Cai et al. 2020).

A recently described salt-tolerant exo-β-1,3-glucosidase from the xerophilic mold *A. chevalieri* was heterologously expressed in *A. oryzae* and was active against laminaribiose and laminarin (but not on cellobiose) and, therefore, may be used in the saccharification of marine biomass (Senba et al. 2023b).

Other salt-tolerant glycosidases have also been characterized including a chitinase from the marine fungus *A. fumigatus* df673 (the truncated chitinase Δ30AfChiJ was heterologously expressed in *Escherichia coli*; He et al. 2022) and a β-galactosidase from the halotolerant *A. tubingensis* GR1 (Raol et al. 2015). Furthermore, the genome of *A. niger* GH1 (a xerophilic mold collected in the Mexican semi-desert) harbors a β-fructofuranosidase or invertase (EC 3.2.1.26) gene, which was heterologously expressed in *P. pastoris*, and whose enzyme product could be used in inverted sugar production (Veana et al. 2014a). It is noteworthy that the invertase produced by *A. niger* GH1 strain can be produced in good yields on cheap substrates such as molasses and sugarcane bagasse (Veana et al. 2014b).

Halophiles have been reported to produce other industrially important glycosidases as well including α-amylases (*A. gracilis*, Ali et al. 2014b; A*. penicillioides*, Ali et al. 2015), pectinases (*A. niger* and other *Aspergillus* spp., Kutateladze et al. 2009), and xylanases (*A. niger* and other *Aspergillus* spp., Kutateladze et al. 2009; *A. gracilis* and *A. penicillioides*, Ali et al. 2014b). More recently, a halotolerant *A. niger* isolate from the Iko River Estuary, South-South Nigeria, was shown to produce halostable amylase, cellulase, and mannanase (Ufot etal. 2022).

#### Other enzymes: lipases, tannases, oxidoreductases

Fungal lipases are widely used in many segments of the biotechnological industry (Singh and Mukhopadhyay 2012). *Aspergillus*-derived lipases have also found their applications in cheese-ripening and synthesis of chemicals (Chandra et al. 2020). Although lipase production by halophiles seems to be an understudied field, lipase production by *A. sydowii*, A*. gracilis*, and *A. restrictus* has been reported (Elwan et al. 1986; Ali et al. 2014a).

Tannases or tannin acyl hydrolases (EC 3.1.1.20) are esterases which decompose digallate to gallate and galloylglucose esters like tannic acid to gallate and glucose. Tannases are used to degrade harmful tannins in tannery effluents (Sutaoney et al. 2024), to provide pharmaceutical industry with gallic acid in the synthesis of thrimethoprim (an antibiotic, Sutaoney et al. 2024), and to improve the sensory characteristics of fruit juice, wine, beer, and tea as well as the taste and digestibility of feed and food (Sahu and Parihar 2021). *Aspergillus* is a well-known tannase-producing genus and, for example, *A. sydowii* tannase-acyl hydrolase has a biotechnological potential in the hydrolysis of coir waste residues (Albuquerque et al. 2020). Importantly, the xerophilic mold *A. niger* GH1 produces a novel tannase under solid-state conditions (Mata-Gómez et al. 2009), which has also been expressed in *P. pastoris* (Fuentes-Garibay et al. 2015).

Xenobiotics cover a remarkably wide array of harmful environmental pollutants, and some of them can be effectively degraded by lignin-modifying enzymes including peroxidases and laccases (Dhagat and Jujjavarapu 2022). Although white rot fungi are well-known lignolytic enzyme producers and, hence, are excellent tools for bioremediation of xenobiotics (Torres-Farradá et al. 2024), peroxidase and laccase producer halophilic Aspergilli (Bonugli-Santos et al. 2010; González-Abradelo et al. 2019) may also find their future applications in mycoremediation technologies under high-salinity conditions, including downstream processing of various industrial wastewaters, e.g., from biorefineries (González-Abradelo et al. 2019).

### Secondary metabolite production

#### Secondary metabolites for pharmaceutical and dermocosmetic industries

Extremophilic fungi including xerophilic and halotolerant or halophilic Aspergilli represent an untapped treasure box of potential drug active ingredients for versatile pharmaceutical and medical applications (Corral et al. 2020; Ibrar et al. 2020; Ibrahim et al. 2023). These *Aspergillus* spp. have mostly been isolated from solar salterns, salt fields, and deserts, and can produce a wide array of cytotoxic compounds with anticancer potential like aspochalasins (*A. flavipes*; Zhou et al. 2004), epicochalasines (*A. flavipes*; Zhu et al. 2016), chytochalasins and rosellichalasin (*Aspergillus* sp. F1; Xiao et al. 2013), terrequinone A (*A. terreus*; He et al. 2004), variecolorquinones (*A. variecolor*; Wang et al. 2007), indole-3-ethenamide (*A. sclerotiorum*; Wang et al. 2011a), and ( +)-terrein (Zhao et al. 2016). A new anthraquinone, rubrumol, produced by *A. ruber* (reported as *E. rubrum*), possessed topoisomerase I inhibitory activity but no significant cytotoxic activities against human cancer cell lines (Zhang et al. 2017).

Other Aspergilli produce antimicrobials such as terremides (*A. terreus*; Wang et al. 2011b) and bisvertinolone (*A. versicolor* [reported as *A. protuberus*]; Corral et al. 2018) as well as antioxidants like eurotinones (*A. variecolor*; Wang et al. 2007), 2-hydroxycircumdatin (*A. ochraceus*; Cui et al. 2009), and antioxidants from marine alga-derived *A. wentii* (Li et al. 2014). Antimicrobial and antioxidant effects of fermentation broths of some *Aspergillus* spp. from solar salterns have been demonstrated but the antibiotics and antioxidants behind these affects have remained yet to be isolated and characterized (Ali et al. 2014a; Wingfield et al. 2023). Antimicrobial features of supernatants of saltpan fungi including *Aspergillus* spp. in Botswana were also demonstrated (Lebogang et al. 2009).).

Interestingly, the halotolerant *A. flocculosus* PT05-1 strain produced ergosteroids, which showed weak cytotoxicity against tumor cell lines and also antimicrobial activity against *Enterobacter aerogenes*, *Pseudomonas aeruginosa*, and *Candida albicans*, and a new pyrrole derivative with antibacterial effect on *E. aerogenes* (Zheng et al. 2013).

It is worth noting that *A. chevalieri* extracts showed high antioxidant and UV photoprotective capacities, which might be attributed to the echinulin and neoechinulin A production of the fungus. These advantageous features may be exploitable in the dermocosmetic industry, e.g., in various sunscreen products (Calado et al. 2021).

#### Optimization of secondary metabolite production

Optimization of secondary metabolite production by these Aspergilli may face special challenges set by the source and subculturing of the strains, subtle variations in experimental protocols, and the often unpredictable and ambiguous effects of osmotic and saline treatments (Overy et al. 2017).

In spite of these difficulties, Zhao et al. (2016) successfully optimized ( +)-terrein production by the salt-tolerant fungus, *A. terreus* PT06-2, using two level Plackett–Burman design and response surface methodology methods.

In a more recent study, the production of aspochalasin D with anti-cancer, anti-bacterial, and anti-fouling biological activities was increased 18.5-fold in *A. flavipes* by the combination of culture condition optimization with a single-factor experiment and response surface methodology and metabolic engineering. The letter approach included two steps: (i) blocking the formation of aspochalasins P and Q via eliminating the shunt gene *aspoA* and overexpressing *aspoG* encoding a pathway-specific transcription factor (Yang et al. 2023).

Furthermore, an ATP sulfurylase (encoded by the *sC* gene) and DNA ligase IV (product of the *ligD* gene) defected, selenite-resistant *ΔligD ΔsC A. chevalieri* double mutant strain has been constructed with increased gene targeting efficiency, which will make the genetic analysis of the osmophilic fungus *A. chevalieri* more efficient (Hiramatsu et al. 2023). Effective transformation methods for the genetic modification of the osmophilic fungi *A. glaucus* and *A. wentii* are also available (Király et al. 2020a; Bodnár et al. 2023).

### Bioremediation in high-salt environments

Halophilic and halotolerant microorganisms are indispensable agents when bioremediation technologies are developed for highly saline soils and wastewaters (Jain et al. 2021; Wang et al. 2023). Importantly, a plethora of filamentous fungi including the Aspergilli can remove harmful wastewater toxicants including heavy metals, dyes, agrochemicals, pharmaceuticals, endocrine disrupting chemicals, hydrocarbons, detergents, etc. (Dasgupta et al. 2024). Concomitantly, mycoremediation strategies can also provide us with many value-added products including enzymes, organic acids, fungal biomass proteins, pharmaceuticals, and biofuels (Dasgupta et al. 2024).

#### Textile dyes

Industrial dyes are among the major environmental pollutants, which are toxic, carcinogenic, and even disrupt ecosystems (Shindhal et al. 2021). Some halotolerant *Aspergillus* species including *A. fresenii* (reported as *A. sulphureus*; Da Silva et al. 2008) and *A. lentulus* (Kaushik and Malik 2010) have been characterized by excellent textile dye decolorizing activities and can therefore be considered in the mycotreatment of dye-bearing wastewaters.

#### Xenobiotics

Mycoremediation of harmful organic xenobiotics by various fungi including *Aspergillus* spp. is an intensively studied area in environmental technology (Akhtar and Mannan 2020; Akpasi et al. 2023). For example, some *Aspergillus* species have been reported to degrade polycyclic aromatic hydrocarbons (PAHs) effectively including marine-derived fungi (Navina et al. 2024). The halophilic *A. sydowii* (EXF-12860) and *A. destruens* (EXF-10411) effectively eliminated both PAHs and pharmaceutical compound via biodegradation-based and bioadsorption-based processes in the presence of 1.0 and 1.9 M NaCl, respectively (González-Abradelo et al. 2019). It is worth noting that *A. atacamensis* EXF-6660 (isolated from a salt water-exposed cave of the hyperarid Atacama Desert, Chile), which is a remarkably chaotolerant, kosmotolerant, and xerotolerant fungus, metabolized versatile organic molecules under saline conditions and removed many xenobiotics with high efficiency including biphenyls, diphenyl ethers, different pharmaceuticals, phenols, and polyaromatic hydrocarbons from wastewater biosolids (Moreno-Perlin et al. 2023).

#### Chloride ion removal from wastewater

Processing of crustacean shells results in ultrahigh chloride content wastewaters because of the large hydrochloric acid needs of these technologies. It is therefore noteworthy that over 70% of the Cl^−^-content of shrimp processing wastewater could be removed within 3 days using the mangrove wetland-derived fungus *A. luchuensis* (reported as *A. piperis*; Han et al. 2023).

#### *Aspergillus tubingensis*–based aerobic granular sludge

To counteract the deleterious effects of high salinity conditions (50 g/L ≈ 0.9 M NaCl), *A. tubingensis* pellets were added to improve the stability of the interactions between key functional species of wastewater activated sludge. The resulting *A. tubingensis*–based aerobic granular sludge showed an increased COD and NH_4_^+^–N removal efficiency, stronger biomass retention capacity, higher metabolic activity, and stimulated extracellular polysaccharide production by sludge microbiome (Chen et al. 2021).

### Heterologous expression of stress tolerance genes in industrial yeast and *Aspergillus* strains

#### Expression of stress tolerance genes of osmophilic Aspergilli in baker’s yeast

As shown in Table 1, expressing various genes from osmophilic *Aspergillus* spp. like *A. glaucus* and *A. oryzae* (Liu et al. 2014, 2015; Liang et al. 2015; Li et al. 2019, 2021) was an effective tool to enhance the general stress tolerance of *S. cerevisiae* including salt, drought, and copper stress. This approach may have significant industrial relevance, as yeast cells are frequently exposed to a wide spectrum of environmental stress conditions, including high osmotic pressure and heavy metal contaminations, in the technological processes in which they are used (Attfield 1997; Deparis et al. 2017; Tse et al. 2021; Postaru et al. 2023).

**Table 1:** Fungal salt tolerance genes expressed in plants and other fungi

Donor species	Protein and function	Modified organism	Enhanced stress tolerance	Reference
*A. candidus*	AcGDH NADP(H)-dependent glutamate dehydrogenase	rice (*Oryza sativa* L. cv. *Kitaake*)	drought, alkali stress, oxidative stress	Yan et al. (2021)
*A. glaucus*	L44 (RPL44) ribosomal protein	*S. cerevisiae*	salt, drought, heavy metal (Cu^2+^)	Liu et al. (2014)
		*Magnaporthe oryzae*	salt, draught	
		tobacco (*Nicotiana tabacum* L.)	salt	
*A. glaucus*	CCHA-2142 DUF3431 superfamily protein	*Arabidopsis thaliana*	salt	Fang et al. (2014)
*A. glaucus*	AgRPS3aE ribosomal protein	*S. cerevisiae*	salt	Liang et al. (2015)
		*M. oryzae*	salt	
		*N. tabacum*	salt	
		*A. thaliana*	salt	
*A. glaucus*	AgGlpF aquaglyceroporin	*S. cerevisiae*	salt, drought, heavy metal (Cu^2+^)	Liu et al. (2015)
		*Neurospora crassa*	salt	
		*A. thaliana*	salt, drought	
		soybean (*Glycine max* L.)	salt	Li et al. (2021)
*A. oryzae*	AoD9D1 and AoD9D2 delta-9 fatty acid desaturases	*S. cerevisiae*	salt	Li et al. (2019)

#### *A. nidulans gfdB* may enhance general stress tolerance in osmophilic Aspergilli

The evolutionary loss of *A. nidulans gfdB* (putatively encoding a NAD^+^-dependent glycerol-3-phosphate dehydrogenase) ortholog was hypothetically linked to the appearance of osmophily in two Aspergilli, *A. glaucus* and *A. wentii* (de Vries et al. 2017). Interestingly, when *A. nidulans gfdB* was inserted into the genome of *A. glaucus*, the stress (oxidative, cell wall integrity, and heavy metal stresses) tolerance of the fungus increased in general without affecting its osmophily (Király et al. 2020a; Bodnár et al. 2023). Because *gfdB* is an important element of the oxidative stress and cell wall integrity stress defense systems of *A. nidulans* (Király et al. 2020b), this gene may be an effective tool in future *Aspergillus* industrial strain developments. Nevertheless, the supplementation of *A. wentii* with *A. nidulans gfdB* partially reversed its osmophily (with 28–35% in the presence of 2 M sorbitol); meanwhile, only minor and sporadic improvements in other environmental stress tolerances were recorded. This study shed light on the limitations of the use of *A. nidulans gfdB* as a stress tolerance enhancing tool in osmophilic *Aspergillus* spp. (Bodnár et al. 2023).

## Agriculture-related applications

Xerophilic/halophilic *Aspergillus* spp. have also found their applications in agriculture covering the following areas: (i) biosolubilization of phosphate, (ii) halophile fungi and genes from halophilic fungi in combating abiotic stress, (iii) xerophilic fungi in the feed and food chain and their control, and (iv) biological control by xerophilic Aspergilli.

### Biosolubilization of phosphate

Phosphorus is one of the most important macronutrients for plants. The outstanding rock phosphate-solubilizing activities of some salt-tolerant *Aspergillus* (*A. niger*, *A. japonicus*) and *Penicillium* (e.g., *P. simplicissimum*) strains isolated from wheat rhizosphere soil are accompanied by remarkable heat, pH, salt, and desiccation tolerances, which can be valuable constituents of biofertilizers to ensure crop productivities (Xiao et al. 2011). Furthermore, the salinity-tolerant strain *A. niger* An2 from from a Chinese cabbage rhizosphere soil effectively activated immobilized phosphates in general calcareous, and acidic, as well as saline-alkali soils (Li et al. 2015).

### Xerophile/halophile Aspergilli and their genes in combatting abiotic stress

#### *Aspergillus* spp. mitigating salt stress

Soil salinization is a rapidly increasing worldwide problem negatively affecting agricultural crop production (Khaasanov et al. 2023). According to Yuan et al. (2023), salt-resistant soybean genotypes recruit, among other fungi, *Aspergillus* spp., which mitigate salt stress, promote plant growth, and increase nutrient availability. Endophytic Aspergilli may also ameliorate salt stress in crops like salt-tolerant *A. terreus* in rice and maize (Siddiqui et al. 2022) and *A. awamori* in mung beans (Ali et al. 2021).

#### Transgenes from halophiles

Heterologuos expression of salt tolerance proteins of halophilic fungi may increase the salt and even general environmental stress tolerances genetically modified plants and fungi (Table 1). Heterologous expression of *A. glaucus* genes encoding some ribosomal proteins and aquaglyceroporin seem to be especially promising tools in the engineering of salt stress– and drought-tolerant agricultural crops like soybean and tobacco (Liu et al. 2014, 2015; Liang et al. 2015; Li et al. 2021). Experssion of *A. candidus AcGDH* encoding a NADP(H)-dependent glutamate dehydrogenase in rice increased grain yield under drought stress (Yan et al. 2021).

#### Xerophilic fungi in the feed and food chain and their control

Xerophilic *Aspergillus* species and the mycotoxins produced by them can cause severe economic losses via spoiling low and medium water activity foods, stored goods, and animal feeds (Delcourt et al. 1994; Greco et al. 2018; Dijksterhuis et al. 2019).

#### Mycotoxin production by xerophilic and xerotolerant *Aspergillus* spp.

Foods and food ingredients like spices may be contaminated by xerophilic and mycotoxigenic Aspergilli, e.g., *A. flavus*, *A. niger*, and *A. ochraceus* (Delcourt et al. 1994). Nevertheless, there are versatile ecological niches mycotoxigenic Aspergilli can occupy and there are many biotic and abiotic environmental factors that have an impact on their mycotoxin productions (Pfliegler et al. 2020). *Aspergillus*-derived mycotoxins entering the feed and food chain can adversely affect the health of both domestic animals and consumers (Peles et al. 2019; Ráduly et al. 2020) and, not surprisingly, attract much interest among academics, agricultural and industrial experts, and the public (Pócsi et al. 2020, 2023).

One of the major aflatoxin (AF)-producing molds, the xerotolerant *A. flavus*, responded to water stress in a temperature-dependent manner considering both growth rates and AF yields but, importantly, it still synthesized aflatoxin under water stress triggered by the non-ionic solute glycerol (Abdel-Hadi et al. 2012; Medina et al. 2015). Importantly, very low water activity *a*_w_ values (0.34–0.72) did not support the growth of *A. flavus* in stored peanuts (Xing et al. 2016).

The xerophilic ochratoxin A (OTA) producer *A. ochraceus* showed higher growth rates in the presence of the non-ionic glycerol; meanwhile, supplementation with NaCl resulted in a lower optimal growth rate over a wider osmotic potential range (Ramos et al. 1999). Considering the other OTA producer *A. carbonarius*, the fungus tolerated osmotic stress elicited by high glucose concentrations much better than high NaCl levels but high concentrations of both ionic and non-ionic solutes drastically inhibited OTA productions (Stoll et al. 2013).

#### Control of xerophilic Aspergilli in the feed and food chain

Importantly, various models to estimate optimal growth rates at optimal water activities (*a*_wopt_) and to predict growth/no-growth boundaries have been elaborated for *A. montevidensis* (= *E. amstelodami*), *A. chevalieri* (= *E. chevalieri*), *A. pseudoglaucus* (= *E. repens*), and *A. ruber* (= *E. rubrum*), which are indispensable data to prevent fungal spoilage (Greco et al. 2018).

There is a plethora of data available in the literature about the efficient control of xerophilic fungi by chemicals including surfactants and fungicides (*E. amstelodami*; Beuchat and De Daza 1992), ozone (*E. amstelodami*; Antony-Babu and Singleton 2011), and ethanol (*E. herbariorum*/*A. glaucus*; Deschuyffeleer et al. 2015). Meanwhile, the efficacy of sorbic acid and potassium sorbate was not satisfactory to control the growth of *Aspergillus* sect. *Aspergillus* species (*A. montivedensis*, *A. chevalieri*, *A. glaucus* [= *E. herbariorum*]) in a bakery product analogue with near neutral pH (Marín et al. 2003) potassium sorbate (0.3%) was found to be a suitable preserving agent against xerophilic fungi (*A. flavus*; *A. glaucus*) on a fermented bakery product analogue at pH near 4.5 at all the *a*_w_ values tested (0.8–0.9; Guynot et al. 2005). Ammonium propionate is used in feed as an antifungal preservative and is preferred over propionic acid as the latter component has a pungent smell. However, the higher pH of this buffer results in a reduction of the efficacy of the component. An addition of medium-chain fatty acids in low concentrations to ammonium propionate resulted in a synergistic increase of damage to dormant spores, germinating spores and hyphae of the fungus *A. chevalieri* (Dijksterhuis et al. 2024).

In addition, many plant extracts have been demonstrated to inhibit effectively the growth of xerophilic fungi such as *Elettaria cardamomum* and *Syzygium aromaticum* extracts against the xerophilic tea contaminant strains *A. niger* ML01 and *A. flavus* ML02 (Al-Sohaibani et al. 2011) as well as *Allium* species (*A. sativum* L., *A. cepa* L., and *A. cepa* var. *aggregatum*) extracts against the groundnut oil isolate *A. flavus* MTCC 10680 strain (Murugan et al. 2014). Similar to other food spoilage fungi, cranberry and lingonberry concentrated inhibited the growth of *Eurotium* sp. EE2 in sugar-reduced strawberry-lime fruit spread (Ermis et al. 2015).

#### Biological control by xerophilic Aspergilli

Some xerophilic *Aspergillus* strains were isolated from dry cured ham and dry beef cecina and were investigated for their miticidal activity against the mites *Tyrophagus putrescentiae*, *T. longior*, and *T. casei* (family Acaridae). Two *E. rubrum* (= *A. ruber*) strains (C47 and C49) together with the culture collection strain *Eurotium cristatum* (= *A. cristatus*) NRRL 4222 showed the strongest miticidal activities, which may be exploitable in the biological control of mite infestations in ham and cecina factories (Ortiz-Lemus et al. 2021).

## Conclusions and future trends

Based on the available literature data, we can see and predict the current and future importance of these fungal species in the following:

(i) The industrial need for salt-tolerant and osmophilic *Aspergillus* spp. will increase because they are an exceptionally rich resource of salt-tolerant enzymes and novel, biologically active secondary metabolites. Large-scale screening for these microorganisms is predictable, especially in not-yet-discovered extreme ecological niches.
(ii) New fermentation technologies based on salt-tolerant and osmophilic *Aspergillus* spp. are clearly foreseeable, which require a thorough optimization of fermentation infrastructure and parameters. Some of these species are also candidates for fermentation in low water activity products.
(iii) Breakthroughs in heterologous fermentation systems for the bulk production of salt-tolerant enzymes are also predictable.
(iv) Salt-tolerant and osmophilic Aspergilli and their stress response genes as transgenes will be promising tools in the maintenance and even improvement of the productivity of agricultural crops especially concerning the increasingly worse scenarios of climate change. *Aspergillus* stress response genes as transgenes may also improve the performance of industrial yeast and *Aspergillus* strains.
(v) Novel control technologies against xerophilic molds are needed to preserve feed and food, to increase food security and reduce waste.
(vi) These fungi may also gain ground as effective biological control agents, e.g., against mites.
